# Acute Severe Mitral Regurgitation Secondary to Ischemic Papillary Muscle Rupture: A Case Report

**DOI:** 10.7759/cureus.13996

**Published:** 2021-03-19

**Authors:** Loba Alam, Glenmore Lasam, Robert Fishberg, David Powell

**Affiliations:** 1 Internal Medicine, Overlook Medical Center, Summit , USA; 2 Cardiology, Mt. Sinai Morningside, Summit, USA; 3 Cardiology, Overlook Medical Center, Summit, USA; 4 Cardiology, Overlook Medical Center, Summit , USA

**Keywords:** acute mitral regurgitation, papillary muscle rupture, transthoracic and transesophageal echocardiography, extracorporeal membranous oxygenation, mitral valve replacement

## Abstract

Mitral valve rupture secondary to ischemic papillary muscle necrosis is rare in the contemporary era due to improved revascularization techniques. However, when it does occur, prompt diagnosis and urgent surgical intervention can be lifesaving. A 69-year-old male with morbid obesity, hypothyroidism, and a family history of coronary artery disease presented to the hospital with chest pain and dyspnea that began five hours prior. He had an acute infero-postero-lateral myocardial infarction due to total occlusion of the left circumflex artery that was revascularized with the deployment of a drug-eluting stent. Two days after the myocardial infarction, the patient had an episode of ventricular tachycardia. He subsequently went into respiratory distress from flash pulmonary edema and developed cardiogenic shock due to acute mitral valve rupture. The patient underwent surgical mitral valve replacement, extracorporeal membranous oxygenation (ECMO), and hemodialysis. His course was complicated by an acute lower gastrointestinal bleed that progressed into multiorgan failure and eventually his demise. This case highlights the need to include papillary muscle rupture high on the differential when evaluating a hemodynamically unstable patient in the setting of an acute myocardial infarction (MI). Rapid diagnosis by urgent bedside echocardiogram and surgical intervention is crucial.

## Introduction

Papillary muscle rupture is a rare and fatal complication of acute ST-segment elevation myocardial infarction in the contemporary age. Rupture of a papillary muscle leads to acute mitral valvular regurgitation with subsequent flash pulmonary edema and cardiogenic shock. A transesophageal echocardiogram can be used to explore the mitral valvular apparatus to understand the mechanism of mitral regurgitation. Prompt recognition and surgical intervention can be lifesaving, although surgical intervention carries high morbidity and mortality risk. We describe the case of an acute severe mitral regurgitation resulting from a posteromedial papillary muscle rupture which occurred within 48 hours of a myocardial infarction.

## Case presentation

**Figure 1:**
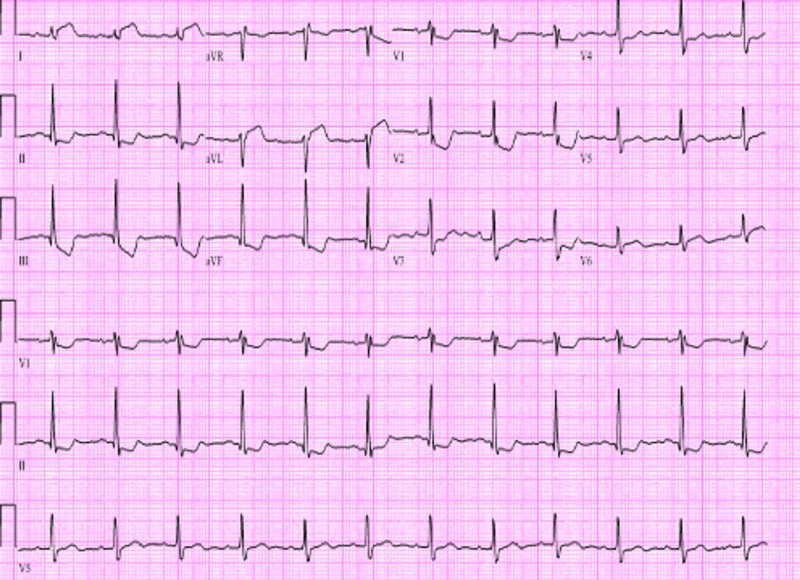
: ECG demonstrated ST-T wave changes in leads II, III, aVF and V1 – V6 suspicious for an acute inferior-posterior wall STEMI. STEMI: ST segment elevation myocardial infarction

A 69-year-old man with a history of morbid obesity, hypothyroidism, and a family history of coronary artery disease presented to the hospital with chest pain and dyspnea which began earlier on the morning of admission approximately five hours prior to hospital presentation. He had a normal cardiopulmonary physical exam and vital signs. Initial labs including hemogram and basic metabolic panel were normal. He had mild troponin elevation of 0.285 ng/mL. He had normal vital signs. Initial ECG demonstrated ST-T wave changes in leads I, aVL, II, III, aVF and V1 - V6 suspicious for an acute infero-postero-lateral wall ST segment elevation myocardial infarction (STEMI) (Figure 1).

**Figure 2:**
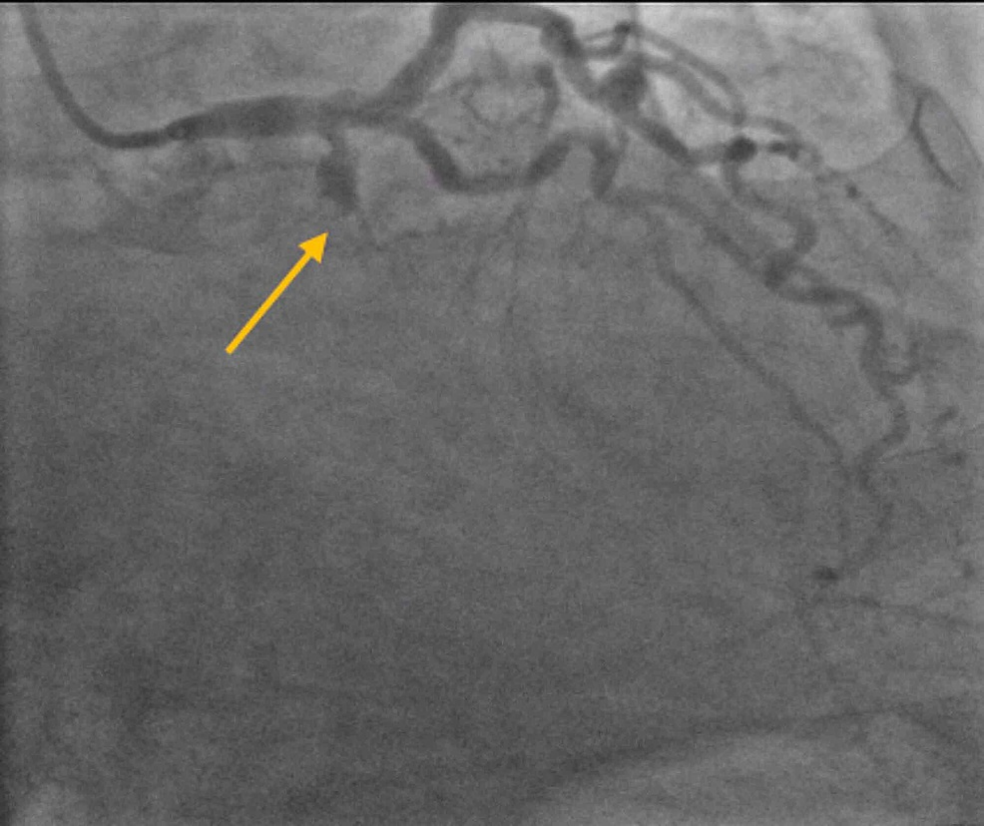
Coronary angiogram revealed a left dominant circulation with a 100% occlusion of the proximal left circumflex artery (arrow).

STEMI protocol was activated, and the patient was brought to the coronary catheterization laboratory emergently. A coronary angiogram revealed a left dominant circulation with a 100% occlusion of the proximal left circumflex artery. The lesion was acute and heavily thrombotic (Figure 2).

The proximal left circumflex lesion was successfully stented with a drug-eluting stent. The left ventriculogram revealed an estimated ejection fraction of 45% with hypokinesis of the inferior and inferolateral segments. The patient was started on aspirin and ticagrelor. 

The patient was steadily improving until post-infarction day two when he developed sudden onset ventricular tachycardia requiring one synchronized cardioversion with conversion to sinus tachycardia. He subsequently developed respiratory distress, hypoxia with oxygen saturation of 85% on room air, and hypotension with blood pressure 80s/50s mmHg, and sinus tachycardia with heart rate in the range of 130-140 beats per minute. At this point, the differential diagnosis included ventricular free wall rupture, papillary muscle rupture, or recurrent myocardial infarction resulting in flash pulmonary edema and cardiogenic shock. 

**Figure 3:**
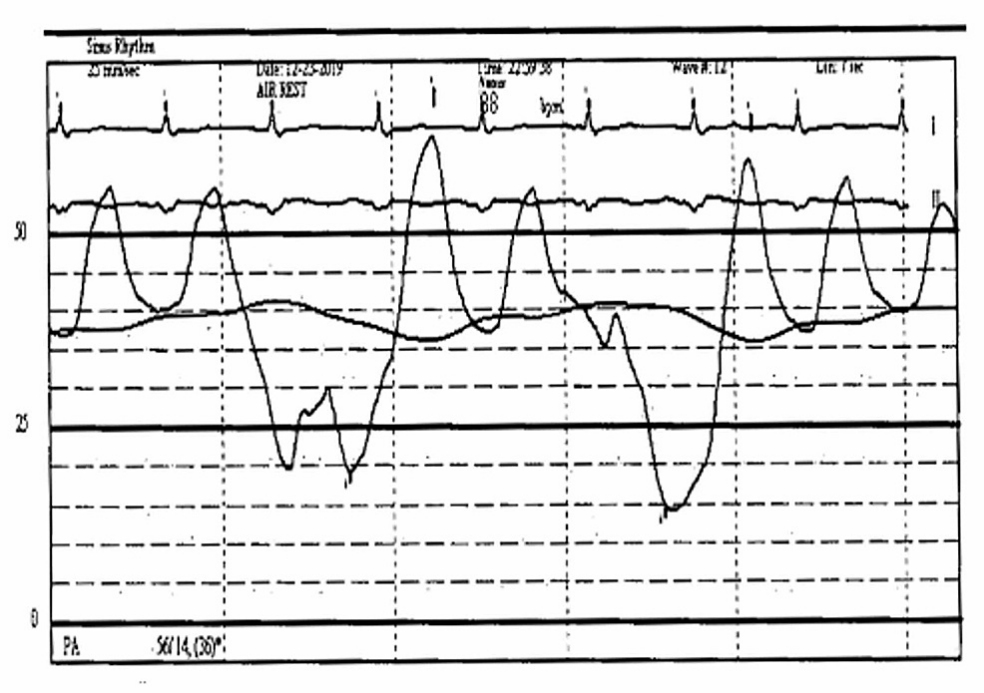
Coronary catheterization revealed severely elevated right heart pressures with pulmonary artery pressure was 60/30 mmHg and a V-wave of 65 mmHg.

An urgent transthoracic echocardiogram (TTE) revealed acute mitral regurgitation (MR), the severity of which was difficult to assess due to tachycardia. Urgent left heart catheterization was performed to exclude acute stent closure. Coronary catheterization revealed a patent stent and otherwise unchanged coronary anatomy. In addition, right heart pressures were severely elevated, suggestive of acute MR and pulmonary edema. The right heart pressures were as follows: Mean right atrial pressure was 30 mmHg, the right ventricular pressure was 55/10 mmHg, the pulmonary artery pressure was 60/30 mmHg, and the pulmonary capillary wedge pressure was 45 mmHg with a V-wave of 65 mmHg (Figure 3).

**Figure 4:**
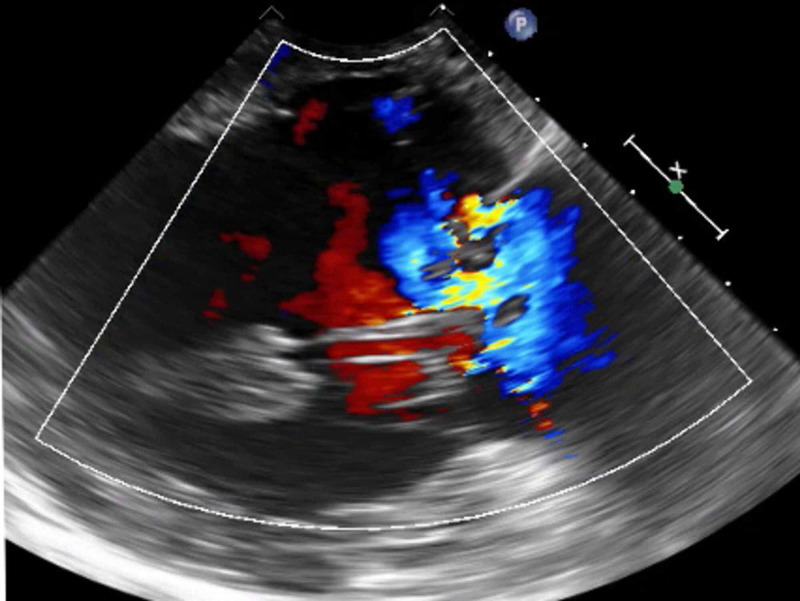
TEE confirmed flail anterior mitral leaflet and chordae due to posteromedial papillary muscle rupture, as well as severe posteriorly directed TEE: Transesophageal Echocardiogram

The cardiac output by the Fick method was 3.1 liters/minute with a cardiac index of 1.7 L/m/m2. After intra-aortic balloon pump (IABP) insertion, the mean pressure was initially 80 to 85 mmHg. Intravenous nitroprusside was initiated, and the patient was intubated due to flash pulmonary edema causing acute hypoxic respiratory failure. Next, an urgent transesophageal echocardiogram (TEE) confirmed flail anterior mitral leaflet and chordae due to posteromedial papillary muscle rupture, as well as severe posteriorly directed mitral valve regurgitation (Figure 4).

The posteriorly directed MR, flail anterior mitral leaflet, and papillary muscle rupture can be further reviewed in supplemental videos (Videos 1- 5). 

**Video 1:** TEE Apical 4 chamber view with color demonstrating posterior directed mitral valve regurgitant flow. TEE: Transesophageal Echocardiogram

**Video 2:** TEE Apical 4 chamber view without color demonstrating flail motion of anterior mitral leaflet. TEE: Transesophageal Echocardiogram

**Video 3:** TEE Deep gastric parasternal long-axis view with color demonstrating mitral valve regurgitant flow. TEE: Transesophageal Echocardiogram

**Video 4:** TEE Deep gastric parasternal long axis view without color demonstrating flail motion of anterior mitral leaflet with papillary muscle rupture. TEE: Transesophageal Echocardiogram

**Video 5:** Apical 2 chamber view without color demonstrating flail motion of anterior mitral leaflet.

Expert Consultation

The patient remained hypotensive despite high dose inotropes and IABP. Cardiothoracic surgery was consulted, and he was emergently taken to the operating room for venoarterial extracorporeal membranous oxygenation (ECMO) placement through a right femoral venoarterial route with percutaneous cannulation. In spite of the very high risk, the decision was made to perform open-heart surgery to replace the mitral valve. 

Final Diagnosis and Treatment 

**Figure 5:**
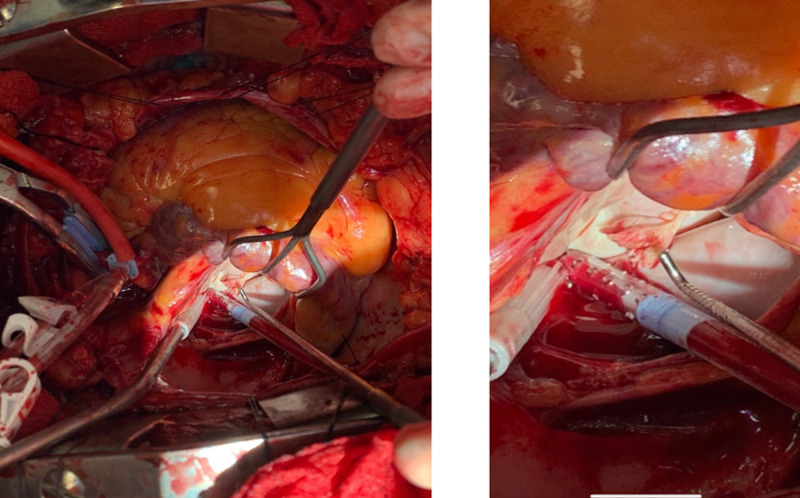
Intraoperatively, the examination of the mitral valve confirmed rupture of the posterior papillary muscle attached to the cord of the anterior mitral leaflet.

Intraoperatively, the examination of the mitral valve confirmed rupture of the posterior papillary muscle attached to the cord of the anterior mitral leaflet (Figure 5). 

The mitral valve was excised and replaced by 33 mm Edwards Lifesciences pericardial tissue heart valve. The cardiopulmonary bypass duration was 147 minutes, after which the patient was once again connected to the ECMO circuit.

Outcome and Follow Up

Postoperatively, the patient required venoarterial ECMO support as well as hemodialysis for five days. Following that, although the patient made slow recovery, his course was complicated by acute lower gastrointestinal bleed that progressed to multiorgan failure. Despite aggressive treatment, the patient expired on postoperative day twelve. 

## Discussion

Acute MR caused by papillary muscle rupture is a rare and life-threatening complication of myocardial infarction. Rupture commonly occurs within five days of an infarct. During this period the cardiac muscle is weakened due to infarction and subsequent necrosis. Acute MR is a cardiac emergency as it presents with sudden onset flash pulmonary edema, hypotension, and cardiogenic shock. Fifty percent of patients with acute moderate to severe MR often have no audible murmur [1]. Severe MR may be silent because reduced ventricular function minimizes the atrioventricular gradient, regurgitant flow, and subsequent murmur [1]. Additionally, the acoustic transmission of the murmur may be obscured by obesity and respiratory distress. 

This case highlights the appreciation of the mitral valve anatomy and its structural integrity. The anterolateral muscle is usually a single large trunk whereas the posteromedial muscle can have one to three heads [2]. Each papillary muscle sends chordae tendineae to attach to both the anterior and posterior mitral valve leaflets. Thus, rupture of either papillary muscle can result in flail of either leaflet. The anterolateral papillary muscle typically has dual blood supply from the left anterior descending and left circumflex arteries. The posteromedial papillary muscle has a singular blood supply from the posterior descending artery which is a branch of the right coronary artery in a right dominant system or the left circumflex artery in a left dominant system. Thus, owing to its singular arterial blood supply, the posteromedial muscle is more susceptible to infarction and subsequent rupture [3]. 

TTE is often the initial modality used to diagnose papillary muscle rupture and it offers a sensitivity of 65% to 85% [4]. However, the mitral valve is a posterior structure, thus TEE offers superior visibility and diagnostic sensitivity of 95% to 100% [5]. The use of color flow Doppler to measure eccentric regurgitant jets improves diagnostic sensitivity in both types of echocardiogram modalities [6]. Once papillary muscle rupture is established, treatment includes emergent surgical intervention with mitral valve repair if the necrosis is limited or prosthetic valvular replacement if the tissue is too friable to be repaired. Concurrent coronary artery bypass graft should be considered as it has shown to improve early and long-term survival [7]. 

While in-hospital mortality rates of surgical correction of acute MR secondary to papillary muscle rupture approach 90% [8], emergent surgical valvular correction approaches a mortality rate of 40% [9]. Low cardiac output states that require ECMO and hemodialysis post-procedure are associated with the poorest outcome [10]. Thus, in the modern era of percutaneous interventions for structural heart disease, an alternate approach to surgery such as transcatheter mitral valve repair could be considered. Indeed, there are isolated case reports where percutaneous MitraClip has been performed to treat acute severe MR in the setting of papillary muscle rupture and cardiogenic shock [11].

## Conclusions

Although with early revascularizations and improved techniques, papillary muscle rupture has become a rare mechanical complication of acute myocardial infarction, physicians should have papillary muscle rupture in the differential when evaluating hemodynamically unstable patients in the setting of acute myocardial infarction. Rapid diagnosis and treatment can be lifesaving. 

Learning Objectives

1. Papillary muscle rupture should be considered in patients with acute decompensation after acute myocardial infarction.

2. The posteromedial muscle is more susceptible to infarction and subsequent rupture due to its singular blood supply from the posterior descending artery.

3. Each papillary muscle sends chordae tendineae to attach to both the anterior and posterior mitral valve leaflets. Thus, rupture of either papillary muscle can result in flail of either leaflet.

4. Rapid diagnosis of papillary muscle rupture by coronary catheterization, TTE, and/or TEE is necessary in order for definitive treatment.

5. Definitive treatment includes traditional surgical repair/replacement, as well as contemporary percutaneous mitral valve repair.
